# Xueshuan Xinmaining Tablet Treats Blood Stasis through Regulating the Expression of F13a1, Car1, and Tbxa2r

**DOI:** 10.1155/2015/704390

**Published:** 2015-03-02

**Authors:** Xiaotian Zhang, Chao Zhang, Jingying Sai, Fan Li, Jinping Liu, Yang Li, Fang Wang

**Affiliations:** ^1^Department of Pathogeny Biology, Basic Medical College, Jilin University, Changchun 130021, China; ^2^Departments of Pharmaceutical, Jilin University, Changchun 130021, China; ^3^Department of Cardiology, First Hospital, Jilin University, Changchun 130021, China

## Abstract

Xueshuan Xinmaining Tablet (XXT), the Chinese formula, has long been administered in clinical practice for the treatment of cerebral thrombosis and coronary heart disease. In this study, we aimed to study the effect and the molecular mechanism of activating blood circulation and removing blood stasis. Rat models of cold coagulation blood stasis were induced with ice-water bath and epinephrine to assess the amelioration of blood stasis by XXT. Microarray technique was used to identify gene expression from the model and XXT-treated rats. In addition, Quantitative Real-Time PCR (qPCR) was performed to verify the microarray results. The results showed that XXT had a good therapeutic effect on blood stasis by reducing the whole blood viscosity (WBV), plasma viscosity (PV), increasing PT, APTT and TT, and by inhibiting platelet aggregation. Genes were differentially expressed in rats among the model group and the XXT-pretreated groups. XXT ameliorated blood stasis by regulating the expressions of F13a1, Car1, and Tbxa2r.

## 1. Introduction

Traditional Chinese medicine (TCM), guided by the theory of traditional Chinese medical science, has been used for over 5,000 years mainly in China and other Asian countries. According to the Chinese medicine theory, body's normal function can be restored by obtaining the balance of* Yin-Yang* of body energy [1]. Chinese medicine's basic principle and key concept include pattern identification, syndrome differentiation, pattern diagnosis, and pattern classification [2]. Blood stasis syndrome (BBS) refers to the circulation of blood that is not smooth, or blood flow that is stagnant and forms stasis [3]. Traditionally, blood stasis relates mostly to chronic diseases and slow-progressing diseases. Recently, many countries have conducted numerous experimental and clinical investigations in various blood stases and obtained some progress. Some “activating blood circulation herbs” were recognized by American medical doctors. Among of these herbs, there basically are the Chinese herbs that invigorate blood and transform blood stasis [4]. BSS is also called Oketsu Syndrome by Japanese medical doctors and Eohyul Syndrome in Korea term, respectively. In contrast to target-oriented Western medicine, traditional Chinese medicines often are composed of an association of individual herbs to form specific formulae to increase therapeutic efficacy and reduce adverse effects [5]. In theory, diverse phytochemical compositions in the TCM formula may simultaneously target multiple molecules/pathways and thus achieve superior effects compared with single compound alone [6]. Xueshuan Xinmaining Tablet (XXT) formula is composed of ten traditional Chinese medicines:* Chuanxiong Rhizoma, Salviae Miltiorrhizae Radix et Rhizoma, Hirudo, Bovis Calculus, Moschus, Pubescent Holly Root, Sophorae Flos, Total ginsenoside of ginseng stems and leaves, Borneolum syntheticum, and Bufonis venenum*. XXT has been widely used on the cerebral thrombosis and coronary heart disease in China for activating blood circulation and removing blood stasis for more than a decade [7, 8]. In spite of the empiric clinical experience, a lack of molecular target weakens the scientific validity of TCM. So new methods for molecular targets are needed to advance the modernization of TCM [9]. Analyzing the changes of gene expression profiles after TCM treatment* in vitro* or* in vivo* may help explain its mechanism of action [10, 11]. In addition, because the use of medicinal herbs may mimic or oppose the effects of concurrently used drugs, gene expression profiles by microarrays may also reveal the mechanism of herb-drug interactions [12]. It is reported that microarray-based transcriptional profiling is used to evaluate TCMs or their components [13–16]. Therefore, we used microarray to explore the possible molecular mechanism and to verify the microarray results by Quantitative Real-Time PCR (qPCR).

## 2. Materials and Methods

### 2.1. Test Article and Treatment

The extract of Xueshuan Xinmaining Tablet (XXT) was provided by Jilin Huakang Pharmaceutical Co., Ltd. Raw materials were purchased from Guanxian (Sichuan), Zhongjiang (Sichuan), Jilin (Jilin), Luoyang (Henan), Tonghua (Jilin), Wenshan (Yunnan), Shijiazhuang (Hebei), Fusong (Jilin), Meishan (Sichuan), and Zhenjiang (Jiangsu), respectively. All of them were authenticated and verified according to the Chinese pharmacopoeia (2010). The voucher specimens (no. HKYY-20130916-20130925) were deposited in the New Drug Research and Development Laboratory of Jilin University. And the preparation method was described as follows:* Salviae Miltiorrhizae Radix et Rhizoma, Chuanxiong Rhizoma, *and* Pubescent Holly Root *were mixed and extracted with eightfold volume of 60% ethanol for 3 hours every time for 3 times. The filtrates were collected and concentrated. Then the concentrated solution was dried with a vacuum and was ground into fine powder (80 mesh).* Sophorae Flos* was decocted in 5-fold volume of water (pH 8~9 adjusted with saturated sodium carbonate solution) for 30 min every time for 3 times. The filtrates were combined and concentrated. Then the concentrated solution was dried with a vacuum and was ground into fine powder (80 mesh).* Hirudo, Moschus, Bufonis venenum*,* Bovis Calculus, Borneolum syntheticum, *and total* ginsenoside of ginseng* stems and leaves were, respectively, ground into fine powder (80 mesh). Finally, all the powder was mixed to get the extract of XXT.

Some standard compounds were also used in this study. Rutin (Lot number: 100080-200707), ferulic acid (110773-200611), salvianolic acid B (111562-200908), Ginsenoside Rg1 (110703-200726), Ginsenoside Re (110754-200822), Ginsenoside Rb2 (111715-201203), Ginsenoside Rb3 (111686-201203), Ginsenoside Rd (111818-201302), Cryptotanshinone (110852-200806), cholic acid (10078-0013), Cinobufagin (110803-200605), Resibufogenin (0718-9004), and Tanshinone IIA (0766-200011) were purchased from the National Institutes for Food and Drug Control (Beijing, China). Ginsenoside Rc was provided by the New Drug Research and Development Laboratory of Jilin University.

**Figure 1:**
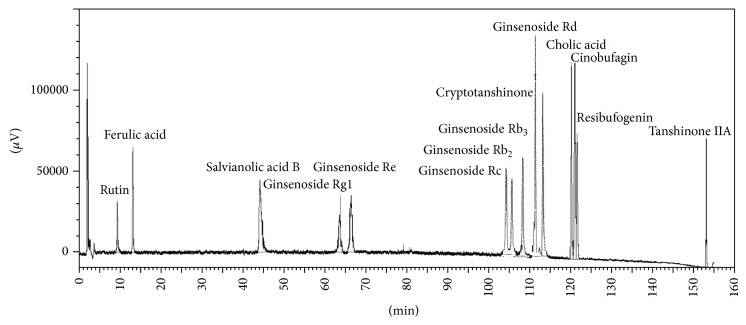
The fingerprints of the mixed standard compounds.

**Figure 2:**
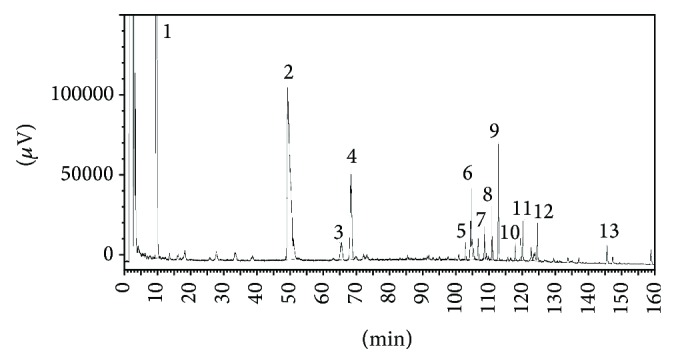
The fingerprints of extract of XXT. (1) Rutin; (2) salvianolic acid B; (3) Ginsenoside Rg1; (4) Ginsenoside Re; (5) Ginsenoside Rc; (6) Ginsenoside Rb_2_; (7) Ginsenoside Rb_3_; (8) Ginsenoside Rd; (9) Cryptotanshinone; (10) cholic acid; (11) Cinobufagin; (12) Resibufogenin; (13) Tanshinone IIA.

The fingerprints of the XXT extract were further analyzed with a high performance liquid chromatography (HPLC) system (Waters, USA) that consisted of a model 1525 Waters pump, model SEDEX FRANCE 75 Evaporative Light-scattering Detector, and Diamonsil C_18_ Column (5 *μ*m, 250 mm × 4.6 mm). The mobile phase was comprised of acetonitrile (A) and 1% acetic acid in water solvent (B). The gradient mode was as follows: initial 18% A linear gradient to 20% A in 20 min; linear gradient to 30% A in 60 min; linear gradient to 37% A in 90 min; linear gradient to 60% A in 105 min; and linear gradient to 100% A in 140 min. The flow rate was 1.0 mL/min. The components of XXT were identified by the comparison of the retention time from the chromatograms with those known standards. The fingerprints of the mixed standard compounds and the extract of XXT were shown in Figures 1 and 2.

Buchang Naoxintong (BN) capsule, produced by Shaanxi Buchang Pharmaceutical Co., Ltd., is another traditional Chinese medicine. It has been approved for the treatment of cerebrovascular and cardiovascular diseases for many years [17]. It has the significant effects of supplementing qi, activating blood circulation, and removing blood stasis [18–21]. Considering the similar and significant effect of Buchang Naoxintong capsules and XXT, we decided to choose it as control drug.

### 2.2. Animals Preparation

Male Wistar rats weighing 180–220 grams (Animal Center of Norman Bethune Medical College of Jilin University, Jilin, China) were purchased one week before the experiment and were habituated to the living and testing environments. The rats were kept under controlled environmental conditions (22 ±2°C, relative humidity 40–60%, 7 am to 7 pm alternate light-dark cycles, and food and water* ad libitum*). The animal protocols were conducted according to the guide for the administration of laboratory animals (Directive 86/609/EEC on the Protection of Animals Used for Experimental and Other Scientific Purposes, 1986) and were approved by the Institutional Animal Care and Use Committee (IACUC) of Jilin University, China.

The experimental groups (*n* = 10) were as follows: (1) normal control (NC), (2) cold- and epinephrine-induced blood stasis syndrome (BSS), (3) BSS model rats with low dose XXT (0.35 g/kg) treatment, (4) BSS model rats with middle dose XXT (0.70 g/kg) treatment, (5) BSS model rats with high dose XXT (1.40 g/kg) treatment, and (6) BSS model rats with “Buchang Naoxintong (BN)” (0.70 g/kg) treatment as positive control. Blood stasis models were made by placing the rats in ice-cold water (0°C~1°C) for 5 min daily for 7 days and by being administrated with two subcutaneous injections of hypodermic epinephrine (1 mg/kg) at 4-hour intervals at the 8th day [22]. Simultaneously, model rats were administered with XXT and BN via gastric irrigation once daily for eight days. The rats in the NC and BSS groups were treated with an equal volume (5 mL/kg) of distilled water as vehicle control. The dose of XXT was chosen based on the clinical application dosage of 2.4 g/day/60 kg body weight. The viscosity, anticoagulation, platelet aggregation, and microarray experiments were performed after the pretreatment of XXT or BN and initiation of the blood stasis.

### 2.3. Model Assessment

To evaluate the success of the BSS model in rats, the hemorheology and coagulation function indexes and platelet aggregation were assessed in this study. The hemorheology indexes of whole blood viscosity and plasma viscosity, the coagulation function index of thrombin time (TT), activated partial thromboplastin time (APTT), prothrombin time (PT) and fibrinogen (FIB), and platelet aggregation were measured according to a previously described method [23, 24].

### 2.4. Viscosity Determination

Rats were anesthetized with 10% chloral hydrate (3 mL/kg) at 17 h at the 8th day after the last injection of epinephrine [23], and blood was drawn from the abdominal aorta for the determination of hemorheological variables. Blood was kept in heparinized (20 U/mL) tubes for whole blood viscosity (WBV) and plasma viscosity (PV) measurements. Plasma was separated from blood by centrifugation at 3000 rpm for 10 min [25] and was stored at −20°C until analysis. The viscosity was determined by a cone-plate viscometer (Model LG-R-80B, Steellex Co., China) at different shear rates at 37°C. The whole blood viscosity was measured with shear rates varying from 1 to 200 s^−1^. Plasma viscosity was measured at high shear rate (200 s^−1^) and low shear rate (50 s^−1^), respectively. All experiments were completed within 3 h after blood collection.

### 2.5. Plasma Anticoagulation Assay

The coagulation function index included thrombin time (TT), activated partial thromboplastin time (APTT), prothrombin time (PT), and fibrinogen (FIB). It was determined by coagulometer (Model LG-PABER-I, Steellex Co., China) with commercial kits, according to the manufacturers' instructions. To establish the standard curve of TT and thrombin concentration, TT was determined by incubating 60 *μ*L of plasma for 3 min at 37°C, followed by the addition of 60 *μ*L of thrombin agent. APTT was determined by incubating 10 *μ*L of the sample solution and 50 *μ*L APTT-activating agents for 3 min at 37°C, followed by the addition of 50 *μ*L of CaCl_2_. PT was determined by incubating 40 *μ*L plasma for 3 min at 37°C, followed by the addition of 40 *μ*L thromboplastin agent and 20 *μ*L of the sample. FIB was determined by incubating 10 *μ*L plasma with 90 *μ*L imidazole buffer for 3 min at 37°C, followed by the addition of 50 *μ*L FIB agent and 10 *μ*L of the sample solution.

### 2.6. Platelet Aggregation Determination

Platelet-rich plasma was obtained after centrifugation of citrated whole blood at 200 g for 15 min, and then platelet-poor plasma was obtained by centrifugation at 2000 g for another 15 min [23]. The aggregation response was measured by the turbidimetric method [26]. After 100% light transmission was calibrated by 300 *μ*L platelet-poor plasma solution, platelet aggregation was induced by the addition of ADP (final concentration: 5 *μ*M) into 300 *μ*L sample of platelet-rich plasma, and the changes in light transmission were recorded.

### 2.7. Statistical Analysis

All quantitative data were given as means ± SD performed using the SPSS 11.5 software package for Windows. Multiple comparisons among groups were performed by one-way analyses of variance (ANOVA). Student's* t*-test was performed by comparing two groups.* P* values less than 0.05 were considered statistically significant.

### 2.8. RNA Extraction

Total RNA was extracted from the rat whole blood of each group using Trizol (Invitrogen, Gaithersburg, MD, USA) reagent. Then RNA was concentrated by isopropanol and was purified using the NucleoSpin RNA Clean-up kit (MACHEREY-NAGEL, Germany) following the manufacturer's protocol. RNA concentration was then determined using Epoch (Bio Tek, Winooski, USA) with A260/A280 ratio between 1.8 and 2.0 and the RNA concentration was adjusted to 1 *μ*g/*μ*L. Agarose gel electrophoresis was used to check the RNA integrity. The RNA samples were stored at −80°C before processing for microarray analysis.

### 2.9. Microarray Processing

The quality of RNA was checked using the Epoch microvolume spectrophotometer system and formaldehyde modified gel electrophoresis. Only high quality RNA (RNA Integrity Number (RIN) > 9.0) was used for microarray experiments. The rat genome oligonucleotide set was generated using 27K Rat Genome Array (CapitalBio's prespotted high-density pairwise oligonucleotide microarrays, version 3.0 CapitalBio Corp., Beijing, China). Each 27K Rat Genome Array consisted of 26,962 amino acid modified 70-mer probes that represented 22,012 genes and 27,044 transcripts. The cRNA synthesis and labeling were carried out following GeeDomc RNA amplification tag protocol. Total RNA from each sample, along with poly A spikes (labeling control), was converted to double-stranded cDNA. Biotinylated cRNA was synthesized by* in vitro* transcription using T7 Enzyme Mix, and then the cDNA was purified with RNA Clean-up Kit (MN). For each sample, 5 *μ*g cRNA was inverse-transcribed using CbcScript II and Random Primer and then purified with PCR NucleoSpin Extract II Kit (MN). The cDNA was labeled with KLENOW using Random Primer. The labeled product was purified with PCR NucleoSpin Extract II Kit (MN). The biotinylated DNA was hybridized to 27K Rat Genome Array overnight at 42°C. Following hybridization, arrays were washed and stained. The signal intensity measurement was performed using LuxScan 10K Microarray Scanner (CapitalBio Inc.). The microarray procedures were carried out according to the manufacture's instruction.

### 2.10. Analysis of Microarray Data

Raw microarray data were first normalized and the bad spots (misprinted spots) were discarded. Genes with signal intensity (Cy3 or Cy5) over 800 were regarded as the expressed ones. Significant changes in gene expression (*P* < 0.05) induced by blood stasis modeling and XXT treatment were then analyzed using the* t*-test software package (provided by CapitalBio). Finally, at least 2-fold-fluctuation in the normal control group was set as differential expression. A Perl program was used to select the differentially expressed genes that returned to the normal level after XXT treatment. In order to reveal changes in gene expression of biological important subsets, microarray data were subject to hierarchical clustering (Cluster 3.0). These genes were clustered into genes upregulated or downregulated in the blood stasis model and the XXT treatment groups.

### 2.11. Quantitative Real-Time PCR Procedures and Data Analysis

Three genes (>2-fold change) that had antithrombotic function were verified by qPCR technique. PrimeScript RT Master Mix (Takara, Beijing, China) was used to obtain the cDNA. qPCR was performed according to the instructions of SYBR* Premix Ex Taq* II quantitative augmentation reaction system (Takara). The reaction was incubated in an ABI PRISM 7300 Fast Real-Time PCR System at the condition of SYBR* Premix Ex Taq* II quantitative augmentation reaction system (Takara). The primer sequences used for Real-Time RT-PCR were shown in Table 1, and all primers were synthesized by Sangon Biotech Company (Shanghai, China).

## 3. Results

### 3.1. The Effect of XXT on Viscosity

The effects of XXT with different dosages on WBV and PV are shown in Table 2. The model rats had a significantly higher WBV and PV than NC. WBV and PV in the 700 mg/kg and the 1400 mg/kg XXT groups were significantly decreased compared to the model group (*P* < 0.01), and the BCN group exhibited a significant decrease in plasma viscosity (*P* < 0.01) but a less significant one in WBV.

### 3.2. The Effect of XXT on Plasma Anticoagulation

The effects of XXT on blood coagulation were evaluated by assays of APTT, PT, TT, and FIB content in the plasma. The level of FIB was increased, APTT and TT were shortened, and PT was significantly decreased in the model rats. The levels of PT, APTT, and TT were increased by XXT (700, 1400 mg/kg) and BCN treatment (*P* < 0.05, *P* < 0.01) as shown in Table 3.

### 3.3. The Effect of XXT on Platelet Aggregation

Platelet aggregation rate in the model group was significantly increased. As shown in Table 3, XXT of 700 and 1400 mg/kg markedly inhibited platelet aggregation compared to the model group (*P* < 0.05, *P* < 0.01). The platelet aggregation percentage in BCN group was also significantly decreased compared to the model group (*P* < 0.01).

### 3.4. Microarray Results and Hierarchical Clustering Analyses

Data fluctuation by over 2.0-fold was considered to be differential expression. After blood stasis modeling, 435 genes were detected with significant change as compared to the normal control, of which 373 (approximately 85.75%) were upregulated. However, 501 differentially expressed genes, of which 307 genes were upregulated, were detected in the XXT group.

**Figure 3:**
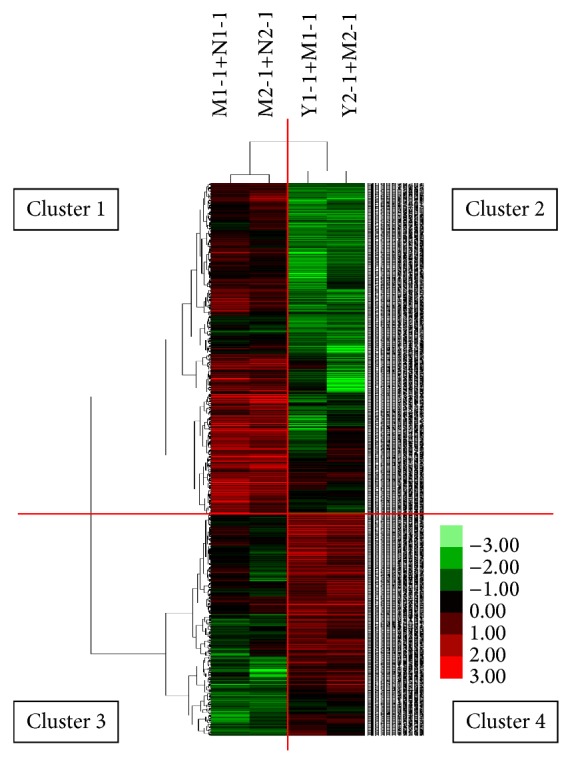
Hierarchical clustering of the differentially expressed genes. Red color indicates a minimum of two fold's increase in expression; green represents a minimum of twofold's reduction in expression. In the left column, two cluster arrays indicate gene expression from the model group versus the normal control (NC) group, and in the right column, the XXT versus model group.

We then conducted hierarchical clustering of the differentially expressed genes (Figure 3). The two main gene clusters were identified visually based on the signal intensity of heat map among different groups. Cluster 1 showed the genes activated by blood stasis. Pretreatment with XXT reduced these gene expressions when compared with the model group, which was shown in cluster 2. In contrast, cluster of genes suppressed by blood stasis (cluster 3) was activated by XXT pretreatment (cluster 4).

### 3.5. Functional Study of the Differentially Expressed Genes

Perl analysis was performed for the gene patterns changed by XXT. 13 overexpressed genes in the blood stasis model were downregulated by XXT, and 3 lower-expressed genes were upregulated by XXT (Table 4). We conducted literature searching for these genes' functions and found that many genes, including F13a1, Car1 and Tbxa2r, were involved in the blood stasis formation.

### 3.6. Quantitative Real-Time PCR Procedures and Data Analysis

**Figure 4:**
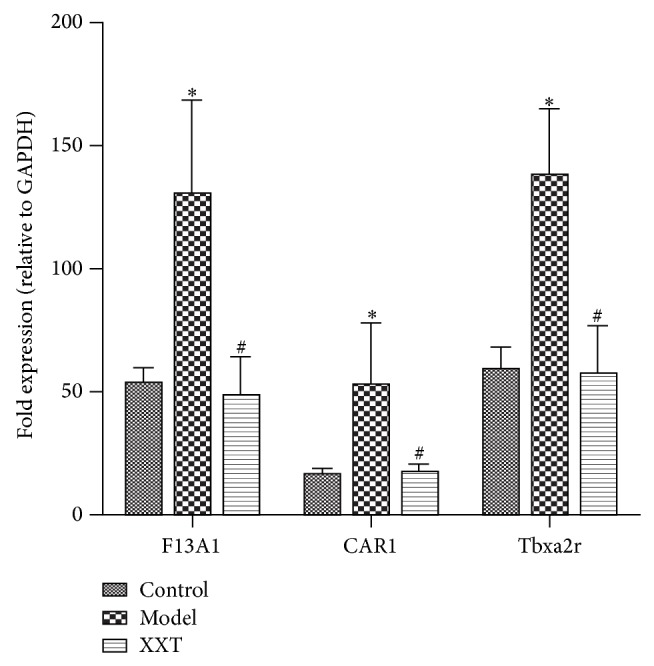
The mRNA expressions of F13a1, Car1, and Tbxa2r. ^*^ *P* < 0.01, the blood stasis model group versus NC group; ^#^ *P* < 0.01, the XXT group versus the blood stasis model group.

The magnitude and direction of changes in expression of the three genes affected by XXT pretreatment were confirmed by qPCR (Figure 4). The expressions of F13a1, Car1, and Tbxa2r were significantly increased (*P* < 0.01) in the blood stasis model but were decreased (*P* < 0.01) to the normal control level by XXT pretreatment. The result demonstrated that abnormal gene expressions associated with blood stasis development could be restored by XXT pretreatment in rat model.

## 4. Discussion

Recently, blood stasis syndrome (BSS) is attached to the theory of promoting blood circulation and removing blood stasis (PBCRBS) by scholars in various countries [27]. In America, doctors are familiar with activating blood circulation herbs (ABC drugs) with functions of promoting blood circulation and removing blood stasis [28, 29]. Blood stasis means the decrease of blood flow velocity, which indicates hemorheological abnormalities [23]. Hemorheological disorders may play an important role in the pathogenesis and development of many diseases. WBV and PV are the two main factors characteristically determining the shear stress upon the vascular wall. Since shear stress is the main stimulus for nitric oxide release [30], it was not surprising that both viscosity are related to endothelium-dependent hemorrheology in platelet aggregation. Here in this study, we showed the model rats had a significantly higher WBV and PV than NC and were significantly decreased by pretreatment with XXT (*P* < 0.01). And the mechanism was investigated by microarray analysis.

Results of microarray technology showed that a multitude of genes was differently expressed in acute blood stasis model rats. These genes, such as gene thrombin receptor-like 2 (F2rl2) and blood coagulation factor XIV (Proc), represent coagulant functions, which are vital in the blood coagulation [31, 32]. To further validate the microarray results, we analyzed a subset of the genes by qPCR. Since blood stasis syndrome was frequently associated with procoagulant, F13a1 and Tbxa2r were quantified by qPCR. F13a1 has been associated with atherothrombotic diseases through changing its levels and activating plasma coagulation factor XIII [33, 34]. F13a1 encodes the subunit of plasma coagulation factor XIII, an essential protransglutaminase involved in hemostasis, especially in the final stages of blood coagulation and the regulation of fibrinolysis [35]. Human thromboxane A2 (TBXA2) induces bronchoconstriction and bronchial hyperresponsiveness, interacts with G protein-coupled TBXA2 receptor (Tbxa2r), and acts as a potent vasoconstrictor and stimulator of platelet aggregation [36]. In the present study, a good correlation of the quantities was demonstrated among the changed transcripts measured by Quantitative Real-Time PCR. We found that F13a1 and Tbxa2r genes were increased in the blood stasis model, and were decreased significantly with XXT pretreatment, which implied that XXT may promote blood circulation and remove blood stasis through preventing plasma coagulation and reducing the activity of G protein-coupled TBXA2 receptor. In addition, we also found that the carbonic anhydrase 1 (Car1) gene was significantly overexpressed in the model group but was reduced to normal level in the XXT pretreatment group. Carbonic anhydrases are zinc metalloenzymes that catalyze the reversible hydration-dehydration of carbon dioxide and bicarbonate [37]. Cars appear to play a role in diverse physiological and biological processes including respiration, acid-base balance, ion transport, bone absorption, renal acidification, gluconeogenesis, and ureagenesis and in the formation of aqueous humor, cerebrospinal fluid, saliva, and gastric acid [38].

## 5. Concluding Remark

Investigating functional mechanism of complex TCM is a challenging task. In this study, the hemorheological and microarray experiments were conducted to explore the anti-blood stasis effect and related mechanism of XXT. The results demonstrated that XXT could significantly reduce the whole blood viscosity, plasma viscosity, increase PT, APTT, and TT, and inhibit platelet aggregation. Moreover, we also found that peripheral blood mRNA expression was dramatically changed in blood stasis model rats. And prophylactic administration XXT could prevent the abnormal expression of genes.

## Figures and Tables

**Table 1:** The primer pairs for the real time PCR.

No.	Gene	Primer sequence	Length (bp)	GC%
1	F13a1-F	CCGAATGCATCGTGGGGAAA	20	55%
	F13a1-R	ACACAGCGTCTTCTTCGCAC	20	55%

2	Car1-F	CAACCAGTCAGTGCTGAAAG	20	50%
	Car1-R	GAACTAAGTGAAGCTCTCCAG	21	47.6%

3	Tbxa2r-F	TGGATGCCCTTGCTGGTCTT	20	55%
	Tbxa2r-R	CGTAGGTAGATGAGCAGTTG	20	50%

**Table 2:** Effect of XXT on the whole blood viscosity (WBV) and plasma viscosity (PV) in blood stasis rats ($\overset{-}{X}\pm s$, *n* = 10).

Group	Dose (mg/kg)	WBV (mPa/s)			PV (mPa/s) 140/s
		20/s	60/s	150/s	
NC	—	9.81 ± 0.45^**^	5.55 ± 0.27^**^	3.76 ± 0.17^*^	0.86 ± 0.05^**^

Model	—	10.45 ± 0.39	6.00 ± 0.30	4.07 ± 0.22	1.22 ± 0.18

XXT	350	10.18 ± 0.54	5.83 ± 0.34	3.87 ± 0.18^*^	0.93 ± 0.04^**^
	700	9.39 ± 0.63^**^	5.33 ± 0.42^**^	3.61 ± 0.28^**^	0.83 ± 0.06^**^
	1400	9.56 ± 0.44^**^	5.44 ± 0.23^**^	3.75 ± 0.13^**^	0.85 ± 0.10^**^

BCN	800	9.65 ± 0.57^**^	5.55 ± 0.47^*^	3.79 ± 0.33^*^	0.86 ± 0.11^**^

^*^ *P *< 0.05, ^**^ *P* < 0.01 versus the blood stasis model. Normal control (NC).

**Table 3:** Effect of XXT on the plasma coagulation parameters and platelet aggregation rate in rats ($\overset{-}{X}\pm s$, *n* = 10).

Group	Dose (mg/kg)	Plasma coagulation parameters				Platelet aggregation rate (%)
		APTT (s)	PT (INR)	TT (s)	FIB (g/L)	

NC	—	21.98 ± 3.44^**^	1.40 ± 0.11^*^	27.42 ± 2.47^**^	1.99 ± 0.13^**^	22.89 ± 5.07^**^

Model	—	16.58 ± 1.61	1.29 ± 0.08	23.68 ± 2.64	4.69 ± 0.40	35.72 ± 3.39

XXT	350	16.94 ± 2.48	1.35 ± 0.09	23.56 ± 2.47	4.57 ± 0.38	31.84 ± 5.45
	700	18.31 ± 2.00^*^	1.39 ± 0.18	27.06 ± 3.35^*^	4.71 ± 0.42	31.06 ± 3.92^*^
	1400	19.52 ± 3.63^*^	1.45 ± 0.23^*^	27.88 ± 3.26^**^	4.54 ± 0.31	28.25 ± 5.42^**^

BCN	800	19.03 ± 3.16^*^	1.37 ± 0.11	26.50 ± 3.07^*^	4.68 ± 0.35	29.65 ± 5.50^**^

^*^P< 0.05, ^**^ *P* < 0.01 versus the blood stasis model. Normal control (NC).

**Table 4:** Information of the genes that change their expression pattern by XXT.

No.	Gene no.	M versus N	XXT versus M	Gene name/description
1	Rn30003360	7.07645	0.38265	—/electron transporter activity
2	Rn30006822	4.32535	0.35525	—/—
3	Rn30004649	65.10415	0.4483	—/—
4	Rn30000949	2.54515	0.2371	—/RGD1563482
5	Rn30018583	2.45385	0.28735	—/runt related transcription factor 2
6	Rn30020887	5.67375	0.35665	Fam58b/—
7	Rn30002724	6.4456	0.21045	—/—
8	Rn30006927	25.1743	0.42055	Dnm2/L25605
9	Rn30006808	4.8359	0.2197	Smtnl1/XM_230278
10	Rn30000910	2.18855	0.35985	—/postmeiotic segregation increased 2
11	Rn30022682	2.98935	0.24595	—/similar to RBT1
12	Rn30004786	27.18685	0.29585	—/idine phosphorylase 2
13	R003120_01	2.6854	0.20565	Slc7a5/tumor-associated protein 1
14	Rn30019843	0.3703	3.41815	RGD1564417/similar to tumor protein D53
15	Rn30007627	0.23865	3.08495	Prg2/proteoglycan 2, bone marrow
16	Rn30014233	0.2301	3.67895	Cdca3/ell division cycle associated 3
